# Factors affecting thyroid nodule fine needle aspiration non‐diagnostic rates: a retrospective association study of 1975 thyroid biopsies

**DOI:** 10.1186/s13044-021-00093-2

**Published:** 2021-02-10

**Authors:** Devon Houdek, Sandra Cooke-Hubley, Lakshmi Puttagunta, Donald Morrish

**Affiliations:** 1 Division of Endocrinology and Metabolism, Department of Medicine, University of Alberta, Edmonton, Canada; 2 Department of Laboratory Medicine and Pathology, University of Alberta, Edmonton, Canada

## Abstract

**Background:**

Thyroid nodules are common in clinical practice, and it is important to distinguish benign nodules, the vast majority, from malignant ones. Non-diagnostic (ND) samples have the potential to delay or mis-diagnose or lead to unnecessary surgeries, and it is important to examine what factors influence the ND rate. Prior literature has suggested that the impact of bedside cytology on ND rate is dependent on the initial adequacy rate, whereby higher ND rates benefit most from bedside cytology. We aim to compare the impact of bedside adequacy review between specialist groups who perform high volume thyroid biopsies with low initial ND rates.

**Methods:**

We reviewed the cytopathology results of 1975 thyroid nodule FNAs performed between January 1, 2017 to December 31, 2017 in a multi-centre Canadian city, and the corresponding histopathology reports of 340 resected nodules. Descriptive variables were used to describe the data along with chi-squared testing and univariate logistic regression.

**Results:**

The FNA biopsies were performed by three different speciality groups, which differed by procedural volume: radiology performed the most at 1171, pathology performed 655 and surgery performed 103. We could not define the operator for 45 of the nodules. The ND rate was lowest in the speciality groups with highest procedural volume, 3.4 % in pathology and 8.3 % in radiology, compared to 37.9 % in surgery (*p* < 0.001). Completion of bedside cytology rapid onsite evaluation (ROSE) significantly reduced the ND rate from 16.7 to 4.2 % for all samples (*p* < 0.001). When ROSE was compared with non-ROSE within a high procedural group (radiology), it further reduced the ND rate from 12.5 to 5.1 % (*p* < 0.001). Of the 340 resected nodules, 10.7 % (18) were in the ND category, of which 28 % (5/18) of these were found to be malignant (4 papillary carcinoma and 1 lymphoma).

**Conclusions:**

The results from this study demonstrate that thyroid FNAs performed with bedside ROSE can significantly reduce the ND rate compared with non-ROSE, even in experienced groups with low initial ND rates. It is therefore imperative that care providers managing patients with thyroid nodules ensure that thyroid FNAs are referred to specialized individuals/groups who do high volume, and ideally with the use of bedside ROSE, whether provided by a cytotechnologist or a pathologist.

## Introduction

Thyroid nodules are very common in clinical practice, with rates as high as 68 % in the general population when high-resolution ultrasound (US) is used for detection. Although the majority are benign, a small proportion (5–15 %) of these nodules harbour malignancies, which include the treatable papillary and follicular thyroid carcinomas and the rarer but more aggressive medullary and undifferentiated thyroid cancers. The American Thyroid Association (ATA) has devised an algorithm for evaluation and management of thyroid nodules based on US pattern and size to recommend fine needle aspiration (FNA) cytology. Briefly, thyroid nodules that have ultrasound features associated with high or intermediate suspicion for malignancy require FNA if size is ≥ 1 cm whereas low suspicion features require FNA ≥ 1.5 cm, and very low suspicion if ≥ 2 cm (observation also an option). This stratification is in place to minimize unnecessary FNAs/thyroid surgeries for nodules that are most likely benign and to avoid over-treating micropapillary thyroid carcinoma which is indolent [1]. In our jurisdiction, patients with thyroid nodules are referred for FNA based on size, or symptoms of compression or other worrisome features, and/or high risk features on imaging. In addition, a case may be referred that may not meet standard criteria in the setting of a known malignancy to determine a primary site or to determine whether the thyroid nodule is benign or malignant/metastasis.

Thyroid nodule FNA biopsy is a relatively simple, cost-effective pre-operative technique used to differentiate between benign and malignant nodules. Cytopathology reports of thyroid FNA are categorized using a universal grading system called The Bethesda System for Reporting Thyroid Cytopathology (TBSRTC), which helps to standardize reporting of diagnostic thyroid cytology results [2]. In the non-diagnostic/unsatisfactory category (Bethesda I), the estimated risk of malignancy is 1–4 %, with usual management a repeat FNA with ultrasound guidance. While the remaining Bethesda categories II-VI show good histopathological concordance, this does not hold true for the ND nodules in the Bethesda I category. A meta-analysis by Bongiovanni et al. (2012) on 6 independent studies found that surgical resection occurred in 16.2 % of ND cases, and amongst these, the risk of malignancy was 16.8 %, much higher than the estimated 1–4 %. They suggested that a ND aspirate obtained by an experienced operator from a sonographically suspicious nodule should be managed cautiously due to the appreciable risk of malignancy [3]. In addition, duplicating these repeat FNA thyroid procedures is not trivial, as it increases patient discomfort, procedural complications and medical costs. Furthermore, a considerable number of patients are lost to follow-up, which could potentially delay or miss a malignancy diagnosis, with resulting adverse patient outcomes [4].

A number of retrospective studies have examined the factors influencing ND rates, which determined that ultrasound guidance, bedside or rapid onsite sample evaluation (ROSE) and operator experience are associated with lower FNA sampling inadequacy [3, 5–9]. Although bedside cytology is an important tool, a meta-analysis by Whitt et al. suggested that the impact of ROSE is dependent on the initial inadequacy rate, and in centres with lower ND rates (due to higher procedural volume or more experienced clinicians), ROSE may not be as beneficial[10]. This study aims to determine whether bedside ROSE can further reduce ND rates of thyroid FNAs amongst experienced, high-volume operators. In addition, we will examine local thyroid malignancies rates based on histopathology of nodules that were resected.

## Methods

We performed a retrospective review of thyroid FNA cytopathology reports using a centralized database, from January 1, 2017 to December 31, 2017, and any subsequent thyroid resection histopathology reports from January 1, 2017 to December 31, 2018. Selection criteria for thyroid FNA biopsies were not available. Individuals younger than 18 were excluded. Reports were accessed via direct data extraction through the Laboratory Information System. The data that was collected included pathology accession number, adequacy of FNA specimen, stated or determined Bethesda category, thyroid resection histopathology results, performing physician specialty, and cyto-histopathology concordance. This project was approved by the University of Alberta Health Research Ethics Board (Study ID: Pro00087370). Descriptive variables were used to describe the data along with chi-squared testing and univariate logistic regression. Thyroid FNA biopsies were performed by ultrasound guidance in the pathology and radiology group. We were not able to determine if this were true in the surgery group. Thyroid FNAs were performed by experienced clinicians in the pathology and the radiology group, with trainees infrequently performing biopsies and only under direct supervision. The pathologists in the pathology group performed bedside ROSE, while in a subset of the radiology group, a cytotechnologist was responsible for bedside cytology. The remaining sub-set radiology group did not have bedside cytology. None of the surgical samples had bedside ROSE. For the groups that performed bedside ROSE, the utility is for checking adequacy of the aspirate only using the diff-quik (air-dried) slides. In this situation, ROSE is not used for bedside diagnosis, and a final cytological diagnosis/report is issued only once the entire case (i.e. all of the slides) are reviewed.

## Results

### Non-Diagnostic Rates by Speciality

A total of 1975 cytopathology reports were reviewed. The thyroid FNAs were performed by one of three speciality groups: radiology completed 1171, pathology completed 655 and surgery completed 103. We were unable to determine who had performed 46 of these samples and these are not included in the comparative analysis. The ND rate of thyroid FNAs was lowest in the two speciality groups (pathology and radiology) which both perform high procedural volume thyroid FNA (> 600 within a group practice) compared the surgical group which performs low volume-FNAs (less than 105 per year). In the high-volume groups, the ND rate was 3.4 % for pathology and 8.3 % for radiology, versus 37.9 % in the low volume surgical group (*p* < 0.001) (see Table 1). Of note, the radiology overall ND rate of 8.3 % was further reduced to 5.1 % with ROSE provided by a cytotechnologist.

**Table 1:** Number of Thyroid Biopsies and Diagnostic Rate by Speciality

	Speciality (n,%)			
	**Radiology**	**Pathology**	**Surgery**	**Unknown**
**Diagnostic**	1074 (91.7 %)	633 (96.6 %)	64 (62.1 %)	36 (88.3 %)
**Non-Diagnostic**	97 (8.3 %)*	22 (3.4 %)*	39 (37.9 %)	10 (21.7 %)
**Total**	1171	655	103	46

*p < 0.001 Pathology vs. Surgery, Radiology vs. Surgery

### Non-Diagnostic Rate by Rapid Onsite evaluation (ROSE) of Cytopathology

Collectively, ROSE was performed on 1320 of the FNA biopsies (68.4 %) procured by two specialities and was provided by cytotechnologists or pathologists. In the pathology group, the pathologist performed both the biopsy and assessed bedside ROSE; in the radiology group, 57 % of the samples had ROSE provided by a cytotechnologist, while the remaining 43 % did not. None of the surgery group FNAs had ROSE.

In the samples which had ROSE (all pathology plus 57 % the radiology samples), the overall ND rate was very low at 4.2 % compared to 16.7 % without ROSE (*p* < 0.001). Within the radiology group, considered a high-volume, experienced group, the total number of FNAs performed with ROSE was 665, while the remaining 506 nodules did not have ROSE. Despite the similar volume and experience of the operators, the group which added bedside ROSE, provided by a cytotechnologist, had a significantly lower ND rate; 5.1 % in samples with ROSE compared to 12.5 % in samples without ROSE (*p* < 0.001) (Table 2).

**Table 2:** Diagnostic Rapid Onsite Evaluation (ROSE)

	All specialities combined (Radiology, Surgery and Pathology)	
	**ROSE**	**Non-ROSE**
**Diagnostic**	1264 (95.8 %)	507 (83.3 %)
**Non-Diagnostic**	56 (4.2 %)	102 (16.7 %)*
**Radiology**		
**ROSE**		**Non-ROSE**
631 (94.9 %)		443 (87.5 %)
34 (5.1 %)		63 (12.5 %) *

*ROSE* Rapid Onsite Evaluation

*p < 0.001 ROSE vs. Non-ROSE, Radiology ROSE vs. Non-ROSE

### Logistic regression

A univariate logistic regression determined that a sample was less likely to be ND if performed by either pathology and radiology compared to surgery (odds ratio [OR] = 0.15, *p* < 0.001, OR = 0.38, *p* < 0.001, respectively). As well, if the sample had bedside ROSE, it was less likely to be ND compared to samples without ROSE (OR = 0.38, *p* < 0.001) (See Table 3).

**Table 3:** Logistic Regression for Variables Affecting Non-Diagnostic Rate

Variable		Co-efficient (SE, 95 % CI)	OR (range)	*P* value
**Speciality**	Pathology	-1.89 (0.37) (-2.63; -1.17)	0.15 (0.07–0.31)	< 0.001
	Radiology	-1.46 (0.24) (-1.9; − 0.9745)	0.23 (0.23–0.58)	< 0.001
	Surgery	Reference	1	
**ROSE**	Yes	-0.97 (0.22) (-1.41 -0.54)	0.38 (0.24–0.58)	< 0.001
	No	Reference	1	

*ROSE *Rapid Onsite Evaluation

### Bethesda Category Rates

The Bethesda category rates for nodules, encompassing all specialities, demonstrated that 8.5 % were Non-diagnostic, 75.9 % were Benign, 4.9 % had Atypia of Unknown Significance/Follicular Lesion of Unknown Significance (AUS/FLUS), 4.3 % were Follicular Neoplasm/ Suspicious Follicular Neoplasm (FN/SFN), 1.11 % were Suspicious and 5.3 % were Malignant (see Table 4).

**Table 4:** Bethesda Categories for all Nodules, Resection Rate and Malignancy Rate

Bethesda Category	n (%)	Resection Rate n (%)	Malignancy Rate n (%)	Pathology
Non-diagnostic	168 (8.5)	18/168 (10.7)	5/18 (27.8)	4 PTC, 1 lymphoma
Benign	1500 (75.9)	120/1500 (8)	5 /120 (4.2)	4 PTC, 1 FTC
AUS/FLUS	96 (4.9)	34/96 (35.4)	4/34 (11.8)	
FN/SFN	85 (4.3)	59/85 (69.4)	13/59 (22)	
Suspicious	22 (1.1)	20/22 (90.9)	17/22 (85)	
Malignant	104 (5.3)	89/104 (85.6)	89/89 (100)	
Total	1975	340	133	

### Histopathology reports

A total of 340 thyroid histopathology reports were identified. The resection rate of ND nodules was 10.7 % (18/168), 8 % for Benign (120/1500), 35.4 % for AUS/FLUS (34/96), 69.4 % for FN/SFN (59/85) and for the Suspicious and Malignant categories were 90.9 % (20/22) and 85.6 % (89/104), respectively. The malignancy rate for the ND category was 27.8 %. In the other categories, malignancy rate was 4.2 % for Benign, 11.8 % for AUS/FLUS, 22 % for FN/SFN and for Suspicious and Malignant categories were 85 % and 100 %, respectively. Amongst the benign samples, there were four papillary thyroid carcinomas and one follicular thyroid carcinoma. Amongst the ND samples there were four papillary thyroid carcinomas and one lymphoma.

## Discussion

### Non‐diagnostic rates by Procedural volume and ROSE

Our study is the first large retrospective study to demonstrate that bedside ROSE statistically decreases the ND rate of thyroid FNA biopsies performed by experienced high-procedural volume clinicians. It is the first study to demonstrate that the value of bedside ROSE is not dependent on initial adequacy rate, and that low ND rates can be significantly lowered by adding bedside ROSE. The key statistic demonstrated is that within a speciality group which performed high-volume procedures (radiology), the ND rate was significantly lower with ROSE than without ROSE, a difference of 7.4 % (5.1 % vs. 12.5 %, *p* < 0.001). Furthermore, this study demonstrates that ROSE may be performed by either a cytotechnologist or pathologist to significantly reduce the ND rate.

Locally, the average FNA thyroid biopsy costs about $400 Canadian. Kuo et al. costed at $394 US or $525 Canadian[11]. The absolute number of reductions in repeat thyroid FNAs can be translated to a cost savings for the hospital system. For example, in our study, we estimate a cost reduction of $12,000-$18,400 Canadian annually, based on performing 30–46 fewer repeat FNAs due to a ND sample. The more repeat biopsies, the higher the additional costs. Although not captured in this data, ND samples can also increase unnecessary surgeries, resulting in further health care costs and potential complications.

A local study published by Isaac et al. (2014) reviewed a total of 180 FNA thyroid nodules, which demonstrated a ND rate of 23 % when performed by Ear/Nose/Throat surgeons[12]. They found that cystic nodules and smaller nodules (< 1 cm) were both statistically more likely to be ND. As well, cystic nodules along with macrocalcifications were found to be predictors for ND results in a study by Choi et al. [6]. Dong et al. (2017) estimated the diagnostic efficacy of ultra-sound guided FNA of 1745 cases of thyroid nodules according to size and US features. They found that diagnostic accuracy was best in nodules sized 5 to 10 mm in diameter, and the false negative rate increased with increasing nodule size (> 20 mm), and false positive highest in the smallest nodules (< 5 mm). However, the majority of these nodules were micronodules (mean size 10.8 +/- 7.6 mm) [13]. Without information on ultrasound characteristics in this study, we were unable to determine whether size or cystic nodules influenced the ND rate. Our assumption is that nodules referred for biopsy were in keeping with the ATA guidelines, and those less than 1 cm were not routinely biopsied. Cystic nodules are associated with low cellularity and had previously been classified as ND. However, the Bethesda Guidelines have since updated recommendations suggested that any sample with abundant colloid should be considered benign, even in absence of the six clusters of follicular cells (i.e. low cellularity). Thus, cystic nodules are less likely to influence ND rates.

A study which reviewed over 4000 thyroid FNA biopsies found experience of the operator influenced the ND rate; within the experienced group (defined as more than 300 thyroid biopsies a year) the percentage of inadequate samples was much lower at 15.4 % compared to 25.8 % amongst the inexperienced group. Bedside ROSE was not performed [6]. DeFliori also demonstrated that high volume and experience reduces ND rate; he tracked a single operator performing thyroid FNAs and found that the ND rate of the first 100 biopsies was highest at 32 %, dropped to 13 % after 200 biopsies and less than 11 % with more than 300 biopsies. Bedside ROSE was not performed in this study [8]. These studies are similar to our findings, in that all three demonstrate that ND rate is a function of procedural volume and experience. However, these prior studies did not evaluate bedside ROSE in the high-volume groups.

A number of studies have examined how bedside ROSE can improve adequacy rates,but have significant limitations to the methodology which confounds the results. Pastorello et al. examined the results of 4649 thyroid FNAs and demonstrated that the diagnostic rate was greater than 90 % with bedside review but dropped to 70 % without (p < 0.0001). In their study, nearly all procedures were performed by radiology residents with assistance from experienced physicians. Therefore, it is difficult to determine if the improved ND rate in the ROSE group is due to ROSE itself or related to the inexperience of the group [14]. A similar finding by Shield et al., which examined results of 3032 thyroid biopsy specimens, showed a ND rate of 6 % with ROSE and 17 % without (p < 0.0001). However, they were not able to quantify training and aspirator experience and suspected that aspirators that performed ROSE were more likely to be more experienced than those who did not [15].

The value of our study is comparing the ND rates in the radiology groups: one with ROSE provided by a cytotechnologist and one without ROSE. Both radiology groups are composed of experienced radiologists, who perform thyroid FNA under ultrasound guidance, and perform over 500 thyroid FNAs per year within the group. Comparing the impact of ROSE within these two subsets of radiology groups allows us to hold constant factors that are lacking in other studies: that is comparing two groups of equivalent experience and procedural volume. We note that the significant reduction in ND rate within the radiology group with ROSE was achieved with the cytotechnologist providing the bedside cytology adequacy; achieving a similar ND rate as the pathologist group. As well, the final cytopathology aspirate in the radiology group were reported by a group of blinded cytopathologists, so that there was no potential bias in reporting the overall adequacy done by the radiologists. This potential bias does exist in the pathologist group in our study as the pathologists who procured the FNA also issued the final cytopathology report on their own samples. We estimate the potential bias of the pathology group is ≤ 2 % (ND rate of radiology with pathologist 5.1 % - ND of pathologist 3.4 %).

### Histopathology of Resected Nodules

A total of 340 thyroid nodules were resected. We were unable to ascertain rationale for surgical excision for Bethesda categories I-III, but this typically depends on patient preference, family history, co-morbidities (concurrent parathyroid surgery for example), or large benign nodules that cause compressive symptoms. The ND category had a resection rate of 10.7 % (18/168), reflecting the uncertainty of this Bethesda category and the malignant potential. A surprisingly high percentage in this category (5/18 or 28 %) was found to be malignant, of which 4 were papillary carcinoma and 1 was a lymphoma. This is much higher than the expected malignancy rate of ND nodules in the Bethesda Category I of 1–4 %, although the literature shows much higher rates of malignancy when these nodules are resected [1]. A meta-analysis of 8 studies completed by Bonviovii found a malignancy rate of 16.8 % in the ND nodules that had been resected and Renshaw et al. reported a malignancy rate of 1–14 % examining the data from over 7000 nodules in a single institution[3, 4]. A small percentage of benign nodules harboured malignancy (4 %), but close to the predicted risk of 0–3 % by the ATA. Within the AUS/FLUS, the malignancy rate was also within the estimated/predicted range (11.8 %, range 5–15 %) as was FN/SFN (22 %, 15–30 %) while Suspicious was a little higher (85 %, predicted 60–75 %) and Malignant was 100 %. The concordance with the Thyroid Cytopathology and Risk of Malignancy within our FNA groups and the ATA recommendations is demonstrative of effectiveness of the Bethesda System for reporting and categorizing cytopathology and the skill and experience of the thyroid surgeons, pathologists and radiologists. Compared to the almost perfect correlation with all other Bethesda categories, the ND category has quite high variability. This variability in malignancy rate in ND aspirates underscores our statement that minimizing ND rates through high procedural volume and bedside ROSE is imperative to appropriately triage thyroid nodules to minimize the risk of unnecessary surgeries and missed thyroid malignancies.

### Limitations

Limitations of the study include comparing the number of thyroid FNAs by speciality group rather than individual physicians. A second limitation was the absence of biopsy techniques (i.e. size of needle gauge, number of passes through the thyroid, aspiration) or characteristics of the thyroid nodule on ultrasound to determine if these characteristics impact ND rate. The type of data available in the administrative database was limited and ultrasound characteristics of the thyroid nodule are not available, nor is needle size or number of needle passes. We had to assume that criteria for FNA biopsy was met *a priori*, and that small nodules (< 10 mm) were not subject to biopsy following recommendations from the American Thyroid Association. The impact of FNA on larger nodules could not be assessed in this study. As well, we were not able to distinguish if the thyroid nodules were repeated biopsies. Finally, selection criteria for FNA thyroid biopsies was not available. Most practitioners use the guidelines from the ATA or American College of Radiology Thyroid Imaging Reporting and Data Systems (TIRADS) to determine if a thyroid nodule requires biopsy, and we had to assume that FNA thyroid nodule referred for biopsy had met ATA or TIRADS criteria.

Strengths of the study included a large sample size with a variety of specialty groups involved which allowed us to perform a real-world comparison of ROSE versus non-ROSE amongst high procedural groups. TBSRTC was used to categorize all the thyroid nodule FNA results, which standardized reporting between groups. As well, follow-up histopathology was available on 340 of the nodules allowing the determination of the ND category malignancy rate.

## Conclusions

In conclusion, we were able to demonstrate that thyroid FNA ND rates can be lowered significantly with high procedural volume within a speciality group, and that bedside ROSE can further reduce the rates, even in high procedure volume specialists. Given that 28 % of the ND samples that were resected harboured malignancy, and other studies having reported similarly high rates, it is essential to minimize the ND rate to avoid missing malignant nodules. Therefore, it is imperative that care providers managing patients with thyroid nodules ensure that thyroid FNAs are performed by trained individuals/groups with high procedural volume, ideally with the use of ROSE.

## Data Availability

The datasets generated and/or analysed during the current study are not publicly available due to this not being a stipulated use of the data in the original study ethics application. They are available from the corresponding author on reasonable request and with permission by the University of Alberta Health Research Ethics Board.

## Abbreviations

FNA: Fine Needle Aspiration.
TBSRTC: The Bethesda System for Reporting Thyroid Cytopathology
ND: Non-diagnostic
ROSE: Rapid Onsite Evaluations
OR: Odds Ratio
TIRADS: Thyroid Imaging Reporting and Data Systems
ATA: American Thyroid Association
